# Aptasensor‐based point‐of‐care detection of cardiac troponin biomarkers for diagnosis of acute myocardial infarction

**DOI:** 10.1002/kjm2.12932

**Published:** 2025-01-03

**Authors:** Tharmaraj Vairaperumal, Ping‐Yen Liu

**Affiliations:** ^1^ Institute of Clinical Medicine, College of Medicine National Cheng Kung University Tainan Taiwan; ^2^ Division of Cardiology, Department of Internal Medicine National Cheng Kung University Hospital, College of Medicine, National Cheng Kung University Tainan Taiwan

**Keywords:** acute myocardial infarction, aptasensor, biomarkers, cardiac troponins, point‐of‐care

## Abstract

Acute myocardial infarction (AMI) represents a critical health challenge characterized by a significant reduction in blood flow to the heart, leading to high rates of mortality and morbidity. Cardiac troponins, specifically cardiac troponin I and cardiac troponin T, are essential proteins involved in cardiac muscle contraction and serve as vital biomarkers for the diagnosis of AMI. Aptasensors utilize synthetic aptamers or peptides with high affinity for specific biomarkers and offer a promising approach for integration into portable, user‐friendly point‐of‐care (POC) applications. This review explores recent advances in POC aptasensor‐based platforms for the rapid detection of cardiac troponin biomarkers. Furthermore, this review addresses current challenges and potential future directions in the development of aptasensor. Also, it highlights their potential to improve timely and accurate diagnosis in clinical and emergency settings.

**Abbreviations**
4‐MBA: 4‐Mercaptobenzoic acid
AMI: Acute myocardial infarction
Au: Gold
AuNPs: Gold nanoparticles
CdS: Cadmium sulfide
CoNi‐MOF: Cobalt‐nickel metal–organic framework
CRP: C‐reactive protein
cTnI: Cardiac troponin I
cTns: Cardiac troponins
cTnT: Cardiac troponin T
CuNWs: Copper nanowires
DTNB: 5, 5′‐dithio bis‐ (2 nitrobenzoic acid)
EGISFET: Field effect transistor with extended gate ion sensitive field effect transistor
Fe₃O₄: Iron oxide
GO: Graphene oxide
HMCS@PDA@AuNPs AuNPs‐loaded polydopamine modified hollow mesoporous carbon spheres: AuNP‐loaded polydopamine modified hollow mesoporous carbon spheres
ITO: Indium tin oxide
MoS₂: Molybdenum disulfide
PB‐AuNPs: Prussian blue‐gold nanoparticles
POC: Point‐of‐care
PtCu DNs: Dendritic Pplatinum‐copper alloy nanoparticles
QDs: Quantum dots
rGO: Reduced graphene oxide
SELEX: Systematically evolved through the exponential enrichment
SERS: Surface‐enhanced Raman scattering
SPCE: Screen‐printed carbon electrodes
SPEECL: Surface plasmon‐enhanced electrochemiluminescence
TAPT‐TDNs: Triple‐aptamer‐modified tetrahedral DNA nanostructures
μPAD: Microfluidic paper‐based analytical device

## 1 INTRODUCTION

Acute myocardial infarction (AMI) is a critical global health challenge, which occurs when there is a sudden reduction or complete blockage of blood flow to the heart, resulting in ischemic damage to myocardial tissue.^1^, ^2^, ^3^, ^4^ AMI, commonly known as heart attack, has become the leading cause of death among young adults.^5^, ^6^ However, most of the research suggests that timely implementation of preventive strategies could effectively prevent nearly 80% of early‐onset cardiovascular conditions.^7^, ^8^, ^9^ Therefore, a timely diagnosis of AMI is critical, as early intervention significantly improves survival rates, reducing the risk of severe complications, and improving patient outcomes.^10^, ^11^

Over the years, cardiac troponins (cTns), specifically troponin I (cTnI) and troponin T (cTnT), have established themselves as gold‐standard biomarkers for the diagnosis of AMI due to their high specificity and sensitivity.^12^ However, conventional laboratory tests used to measure cTn levels are often time‐consuming and require specialized equipment and centralized facilities.^13^ Point‐of‐care (POC) diagnostic tools have emerged as a promising solution to address this gap, offering rapid on‐site testing capabilities that can facilitate timely medical interventions.^14^, ^15^

Aptamers are short synthetic oligonucleotides or peptides that can bind to specific targets with high affinity and specificity.^16^ Their unique properties, including chemical stability, low immunogenicity, ease of modification, and cost‐effective production, position them as a superior alternative to antibodies in diagnostic applications.^17^ Aptasensors, when integrated with advanced transduction platforms, such as optical, electrochemical, or fluorescence‐based systems, enable ultrasensitive and specific detection of cTns, even at low concentrations.^18^, ^19^, ^20^ Among the various POC diagnostic approaches, aptamer‐based sensors, or aptasensors, have gained considerable attention for their potential to transform cardiac biomarker detection.

**FIGURE 1:**
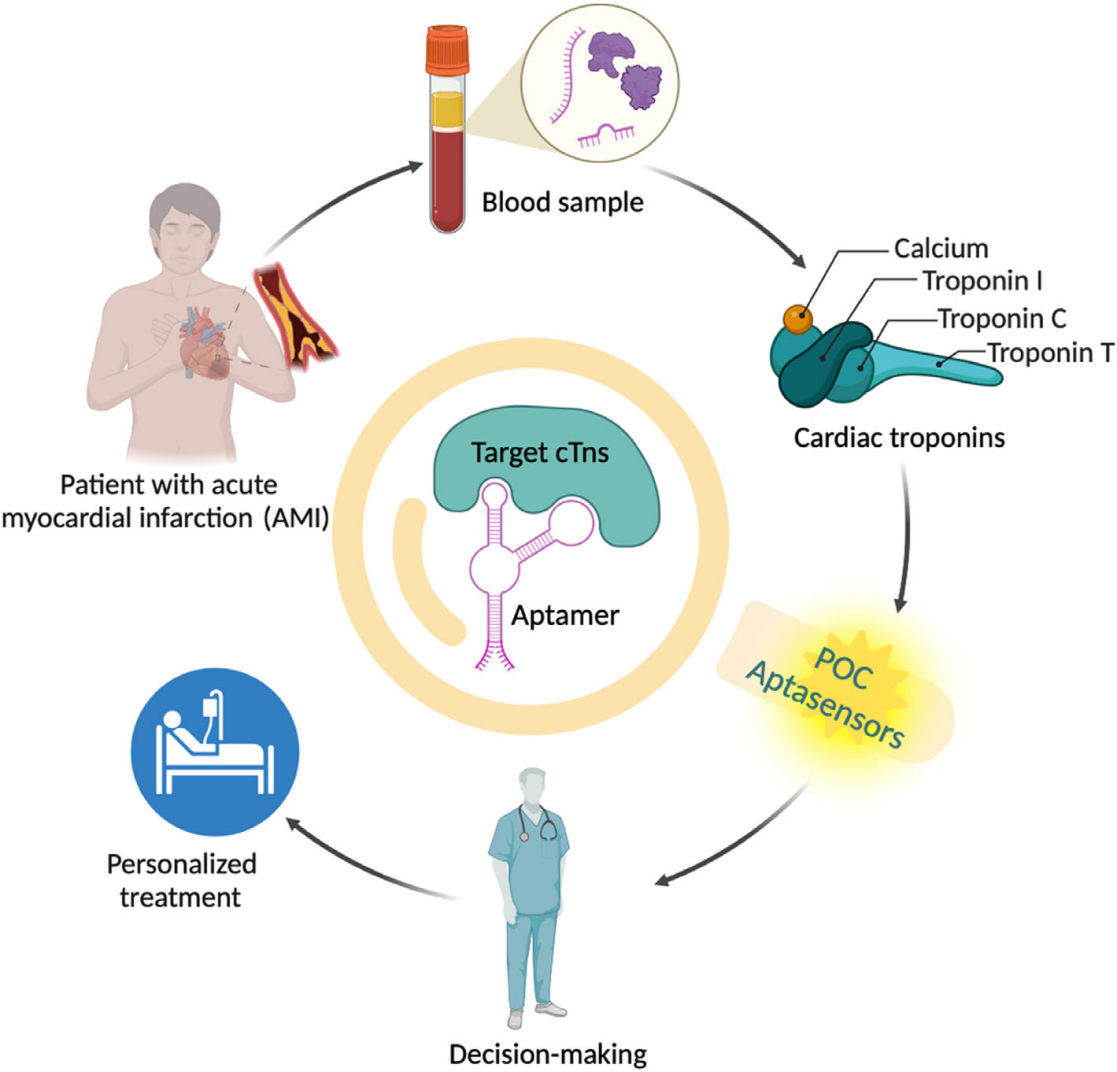
Overview of aptasensor‐based POC detection of cTns for the diagnosis of acute myocardial infarction.

This review provides a comprehensive overview of the structural and functional characteristics of cTns and their diagnostic importance in AMI. Highlight recent advances in aptasensor technologies, focusing on their application in POC settings for detecting cTns. The review also discusses innovations in optical and electrochemical signal transduction platforms that have improved the performance of aptasensors, enabling rapid, reliable, and quantitative analysis. Figure 1 illustrates an overview of a review article on aptasensor‐based POC detection of cTns in the diagnosis of AMI.

## 2 CARDIAC TROPONINS AS BIOMARKERS OF AMI

### 2.1 Structure and function of cardiac troponins

**FIGURE 2:**
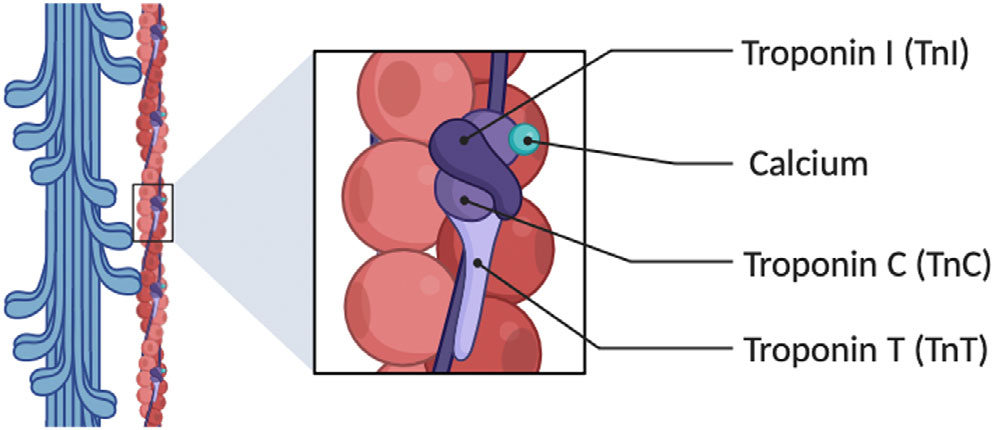
Schematic illustration of the cardiac troponin complex (cTnI, cTnT, and cTnC) and its interaction with calcium.

cTns are structural proteins involved in the regulation of muscle contraction within cardiac muscle cells. The complex contains three subunits, such as troponins I, T, and C, with troponin I and T isoforms unique to cardiac muscle, making them highly specific biomarkers for myocardial injury.^21^ Figure 2 illustrates the structural organization of the troponin complex on the thin filament of cardiac muscle fibers, detailing its interaction with calcium ions. The troponin complex consists of three subunits: troponin I, troponin T, and troponin C. cTnI acts as an inhibitory subunit, responsible for preventing the actin–myosin interaction in the absence of sufficient calcium and thus regulating contraction.^22^ cTnT anchors the troponin complex to actin filaments, ensuring structural integrity during contraction. cTnC binds calcium ions, initiating a conformational change that displaces troponin I, thereby exposing binding sites on actin for myosin and facilitating muscle contraction.

### 2.2 Clinical importance and diagnosis of cTnI and cTnT

During myocardial injury, cardiac myocytes release troponin into the bloodstream as a result of cellular disruption or membrane damage.^23^ The release kinetics of troponin are influenced by the severity of the injury, with levels usually rising within hours after the injury and remaining elevated for days.^24^ cTnI and cTnT have emerged as the gold standard biomarkers for the diagnosis of AMI due to their high specificity and sensitivity in detecting myocardial injury.^25^ These proteins are essential in the regulation of cardiac muscle fiber contraction and are released into the bloodstream upon cardiac cell damage, typically within hours of an ischemic event, thus providing a reliable indicator of myocardial injury.^23^ Numerous studies underscore the clinical utility of cTnI and cTnT in differentiating AMI from other noncardiac causes of chest pain, a critical aspect in emergency care settings.^26^ In addition, elevated levels of these biomarkers are associated with adverse outcomes, which makes them valuable for the stratification of risk and management of patients with AMI.^27^ As such, both cTnI and cTnT have become integral to clinical guidelines for the diagnosis and management of AMI, and advances in POC detection technologies further enhance their applicability in rapid diagnostics.^28^, ^29^

## 3 APTASENSORS

Aptamers, synthetic oligonucleotides carefully selected for their brilliant affinity to specific molecular targets, exhibit remarkable specificity and can be engineered to bind with target molecules.^30^ These aptamers are systematically evolved through the exponential enrichment (SELEX) process, enabling the selection of highly specific binding sequences tailored to a wide range of targets, including small molecules and complex proteins.^31^ Aptasensors are constructed using aptamers, such as synthetic, single‐stranded oligonucleotides, or peptides, that bind to specific target molecules with high affinity.^32^ In recent years, aptasensors have gained significant attention in clinical diagnostics due to their exceptional stability, minimal immunogenicity, ease of chemical modification, and reproducibility.^33^

### 3.1 Aptasensors in point‐of‐care applications

Aptasensors have emerged as a transformative tool in POC diagnostics due to their exceptional specificity, sensitivity, and versatility in the detection of a wide range of biomarkers.^34^ Aptasensors are highly adaptable for POC applications because of their chemical stability, cost‐effective synthesis, and their ability to be easily modified for integration into portable platforms.^35^ Additionally, advances in nanotechnology and signal amplification strategies have significantly enhanced the performance of biosensors, enabling ultrasensitive detection with minimal sample volumes.^36^, ^37^, ^38^, ^39^, ^40^, ^41^, ^42^, ^43^ Aptasensors integrate with optical, electrochemical, or fluorescence‐based transduction systems that offer real‐time and quantitative results, which are crucial for early disease detection, particularly in resource‐limited settings.^44^, ^45^, ^46^ Aptasensors have the potential to revolutionize POC diagnostics by bridging the gap between laboratory testing and healthcare delivery at the bedside. In particular, several POC aptasensors have been developed for the detection of cTn biomarkers in the diagnosis of AMI.

## 4 APTASENSOR‐BASED POC DETECTION OF CARDIAC TROPONINS

Aptasensors‐based POC detection has emerged as a promising tool for POC diagnostics with high sensitivity and specificity, utilizing their compatibility and selective molecular recognition capabilities.^47^ This section explores the recent advances in aptasensor‐based optical and electrochemical signal transduction platforms for rapid and accurate cTns detection in POC settings.

### 4.1 Aptasensors based on optical signal transduction platforms for cTns

**FIGURE 3:**
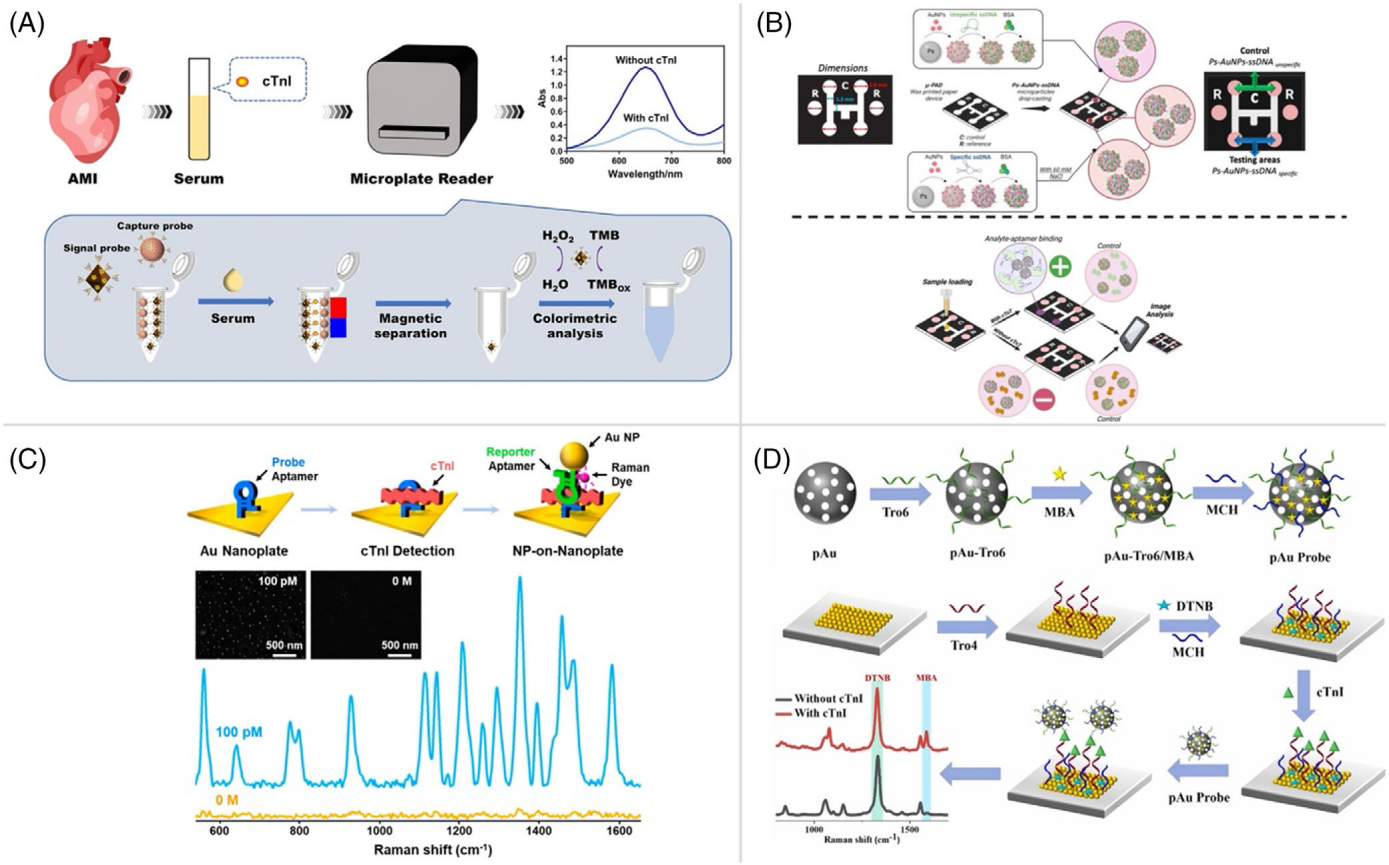
(A) The aptamer‐based colorimetric platform for the detection of cTnI in serum using Fe₃O₄‐Au‐TAPT‐TDNs biosensors highlighting the assembly and detection process of the aptasensor. (Copyright Permission from Elsevier, 2023.) (B) Schematic representation of the design and assembly process of the μ‐PAD device with the cTnT detection mechanism using the μ‐PAD platform. (Copyright Permission from Elsevier, 2023.) (C) Schematic illustrations of the detection of cTnI using an Au nanoplate functionalized with aptamer and Cy5 SERS spectra are shown for AuNPs‐on‐nanoplate structures before (orange) and after (blue) detection of cTnI (100 pM) (Copyright Permission from MDPI, 2020.) (D) The fabrication and detection process of a novel SERS‐based ratiometric aptasensor to quantifying cTnI in human serum samples using an immobilized AuNPs array of Tro4 aptamers modified with sulfhydryl. (Copyright Permission from Elsevier, 2024.)

Recent advancements in optical signal transduction platforms have significantly improved the rapid and sensitive detection of cTns, including cTnI and cTnT, facilitating early and accurate diagnosis of AMI. A colorimetric aptasensor that employs multifunctional hybrid nanoprobes combined with triple‐aptamer‐modified tetrahedral DNA nanostructures (TAPT‐TDNs) has been developed for the detection of cTnI, as shown in Figure 3A.^48^ TAPT‐TDNs demonstrated significantly higher affinity compared to single‐stranded aptamers. This biosensor employs Fe_3_O_4_‐Au‐TAPT‐TDNs as capture probes and MIL‐101 (Fe)‐HRP‐TAPT‐TDNs as signaling probes, forming a sandwich structure for the detection of cTnI based on visible color changes with a limit of 27 pg/mL. This anisotropic aptamer‐mediated approach offers a rapid, accurate, and clinically viable alternative to traditional immunoassays. Similarly, a platform of colorimetric and microfluidic paper‐based analytical device (μPAD) platform was designed for the detection of cTnT during the early stages of AMI (Figure 3B).^49^ Functionalized with gold‐decorated polystyrene microparticles and a colorimetric platform based on aptamer‐specific cTnT achieved dual detection ranges (0.01–0.8 μg/mL and 6.25–50 μg/mL) with an impressive detection limit of 0.0004 μg/mL, further demonstrating its utility in rapid diagnostics. In particular, the study developed a fluorescent aptasensor utilizing a graphene oxide (GO) platform that has been reported for highly sensitive and selective detection of cTnI.^50^ The GO platform quenches the fluorescence of the anti‐cTnI aptamer, which is restored upon cTnI binding as a result of the aptamer's strong affinity for the target cTnI. This method provides excellent analytical performance, with a detection range of 0.10–6.0 ng/mL and a low detection limit of 0.07 ng/mL. Its high selectivity against interfering proteins underscores its potential for the reliable quantification of cTnI in human serum.

Surface‐enhanced Raman scattering (SERS) platforms have also emerged as powerful tools for the detection of cTns. An SERS platform immobilized with aptamer‐gold nanoplate has demonstrated (Figure 3C) precise detection of cTnI with ultralow concentrations of 100 aM (2.4 fg/mL) in buffer and 100 fM (2.4 pg/mL) in serum, with a total assay time of approximately 7 h.^51^ This platform, characterized by atomic force microscopy, exhibited high accuracy, making it a promising candidate for the clinical diagnostics of AMI. Another approach based on SERS involves a ratiometric aptasensor utilizing a gold array substrate modified with the aptamer Tro4 and DTNB for self‐calibrating SERS signals as shown in Figure 3D.^52^ Combined with a porous gold probe containing the aptamer Tro6 and 4‐MBA, this sensor achieves quantification through the SERS intensity ratio (I4‐MBA/IDNB). With a detection limit of 0.27 pg/mL and a linear range of 0.001–100 ng/mL, this platform demonstrated excellent sensitivity, reproducibility, and ELISA‐comparable performance for the diagnosis of AMI. A label‐based SERS strategy utilizing Fe₃O₄@Ag@Au magnetic bimetallic nanoparticles, a specific aptamer, and the Bradford method has further enhanced the sensitivity of cTnI detection.^53^ The Fe₃O₄@Ag@Au substrate exhibited high magnetic properties and uniform signal enhancement, enabling strong Raman responses. Using Coomassie Brilliant Blue G‐250 as a signal reporter, the platform achieved a linear detection range of 0.01–100 ng/mL and a detection limit of 5.50 pg/mL. High recovery rates (92–115%) and low RSD (7.4–12.7%) further validated its reliability for human serum analysis. These advances in optical signal transduction platforms highlight their potential to revolutionize the rapid and sensitive detection of cTns, contributing significantly to the early diagnosis and management of AMI.

### 4.2 Aptasensors based on electrochemical signal transduction platforms for cTns

**FIGURE 4:**
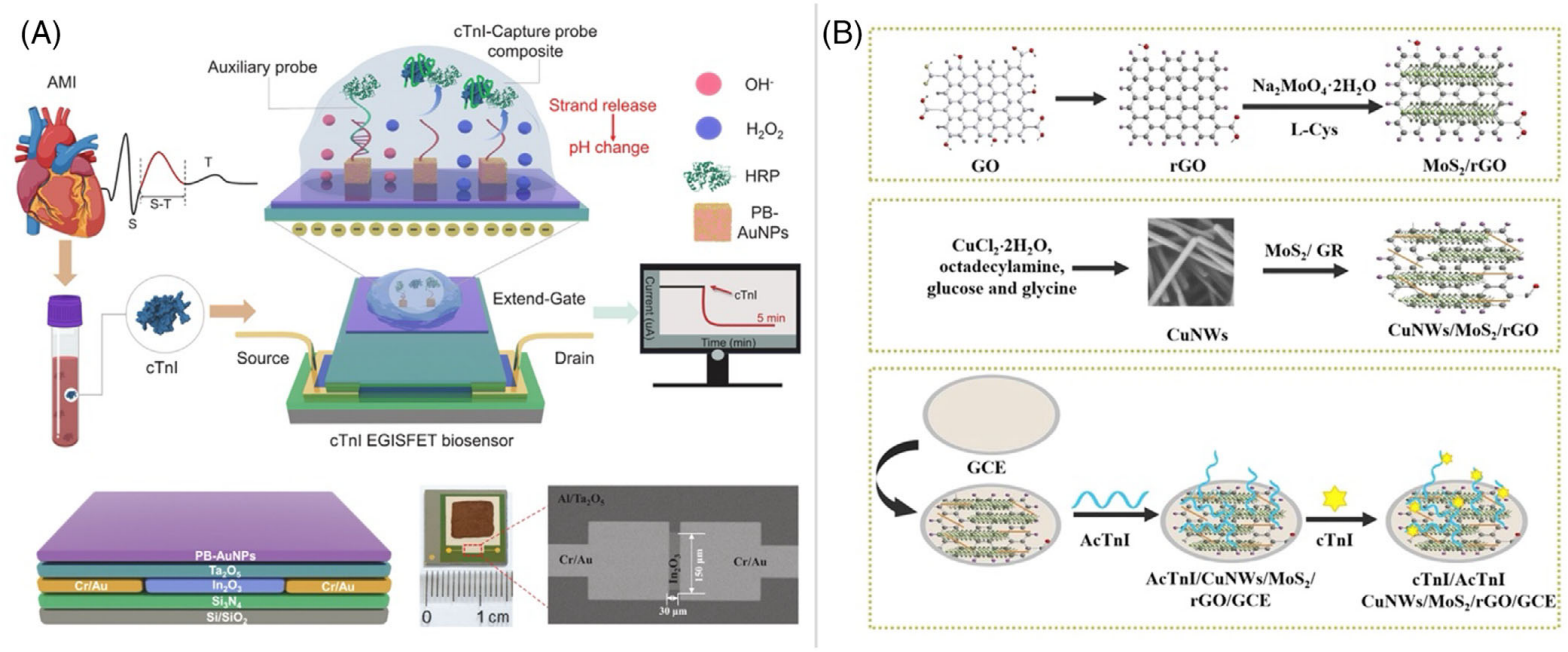
(A) Schematic illustration of the EGISFET‐based aptasensor for rapid detection of cTnI, including the device structure, the digital image of the EGISFET chip, and the morphology of the In₂O₃ channel. (Copyright Permission from Elsevier, 2024.) (B) Schematic illustration of the preparation process for the CuNWs/MoS₂/rGO nanocomposite and the electrochemical sensing strategy for the detection of cTnI. (Copyright Permission from Elsevier, 2021.)

Electrochemical aptasensors have emerged as important diagnostic tools due to their rapid response, high sensitivity, and cost‐effectiveness, making them ideal candidates for POC applications. These platforms are particularly well suited for the detection of cTns, which are key biomarkers of AMI, as they can deliver precise and timely measurements critical for early diagnosis and management in clinical settings. In particular, the study identified single‐stranded DNA aptamers for cTnI using the SELEX method, with the Tro4 aptamer exhibiting exceptional binding affinity (Kd = 270 pM) compared to conventional antibodies (Kd = 20.8 nM). An electrochemical aptasensor utilizing ferrocene‐modified silica nanoparticles and square‐wave voltammetry was developed for cTnI detection.^54^ The sensor demonstrated an impressive linear detection range of 1 to 10,000 pM and an ultralow detection limit of 1.0 pM (24 pg. / mL), allowing it to surpass the clinical cutoff values for AMI. A recent study introduces an ultrasensitive electrochemical aptasensor based on an extended gate ion‐sensitive field effect transistor (EGISFET) that was developed (Figure 4A) for the rapid quantification of cTnI, a key AMI biomarker, in 5 min.^55^ Enhanced sensitivity was achieved using Prussian blue‐gold nanoparticles (PB‐AuNPs) as a signal magnifier and DNA loading platform, coupled with a target‐induced strand‐release strategy for accelerated recognition. The aptasensor demonstrated a wide linear range (1–1000 pg/mL), an ultra‐low detection limit (0.3 pg. / mL), and accurate results in real serum samples. Furthermore, another study introduced a novel sandwich‐type electrochemical aptasensor for the detection of cTnI, using dendritic platinum‐copper alloy nanoparticles (PtCu DNs) in ultrathin graphene‐like CuO‐TiO_2_ nanosheets as the label material and AuNPs‐loaded polydopamine‐modified hollow mesoporous carbon spheres (HMCS@PDA@AuNPs) for electrode modification.^56^ HMCS@PDA@AuNPs enhances electronic conductivity, while PtCu DNs/MUN‐CuO‐TiO_2_ improve catalytic performance and signal amplification. This aptasensor achieved a detection range of 0.01 pg/mL to 500.0 ng/mL with an ultralow detection limit of 2.3 fg/mL, demonstrating excellent sensitivity and reliability in human serum samples and making it a promising tool for the clinical diagnosis of cTnI. In addition, the application of ternary nanostructures, such as copper nanowires (CuNWs), molybdenum disulfide (MoS_2_), and reduced graphene oxide (rGO), has been explored to improve electrochemical activity as shown in Figure 4B.^57^ The aptasensor constructed with CuNWs/MoS_2_/rGO composite material demonstrated excellent selectivity, stability, and reproducibility, with a linear detection range of 5.0 × 10^−13^ to 1.0 × 10^−10^ g/mL and a detection limit of 1.0 × 10^−13^ g/mL. This system successfully detected cTnI in human blood samples, highlighting its potential for the detection and diagnostics of clinical biomarkers.

Interestingly, a rapid multiplexed electrochemical sensor platform has been developed for the simultaneous detection of multiple AMI biomarkers, including cTnT, cTnI, and C‐reactive protein (CRP).^58^ Using nanostructured gold‐modified laser‐scribed graphene (LSG), we integrated aptamer‐based sensors for the selective detection of these biomarkers. The sensor demonstrated detection limits of 1.65 ng/mL for cTnT, 2.58 ng/mL for cTnI, and 1.84 ng/mL for CRP. Clinical validation of blood samples from 51 AMI patients and 9 healthy controls confirmed its precision, with results aligning closely with standard laboratory tests. In another approach, a highly sensitive aptamer‐based sandwich‐type surface plasmon‐enhanced electrochemiluminescence (SPEECL) immunosensor for cTnI detection has been developed that utilizes CdS quantum dots (QDs) as luminophores and AuNPs as plasmon enhancers.^59^ Incorporation of AuNPs amplified the signal by approximately fivefold, leading to a detection range of 1 fg/mL to 10 ng/mL and a detection limit of as low as 0.75 fg/mL. This sensor demonstrated ultra‐sensitive analytical performance, making it a promising tool for highly sensitive cTnI diagnostics. Also, the development of impedimetric aptasensors, such as those utilizing indium tin oxide (ITO) modified with thiol groups and AuNPs, has provided a new avenue for the detection of cTnI.^60^ The Tro6 aptamer selectively binds to cTnI, and impedance changes are measured using a hexacyanoferrate (II)/(III) redox probe. This sensor demonstrated a wide linear detection range (0.1 pg/mL to 10 ng/mL) and an exceptional detection limit of 0.055 pg/mL. Its successful application in human serum samples further validated its potential for clinical monitoring of cTnI. Additionally, an electrochemical sensor utilizing a CoNi‐MOF composite on screen‐printed carbon electrodes (SPCE) demonstrated remarkable stability, reproducibility, and electrocatalytic activity for cTnI detection.^61^ With a detection range of 5–75 pg/mL and a detection limit of 13.2 pM, this cost‐effective sensor is highly versatile, offering significant potential for early detection of AMI and clinical monitoring. Together, these advances in electrochemical aptasensors highlight their transformative potential in the detection of cTn biomarkers. These sensors offer robust solutions for early detection, rapid diagnosis, and continuous monitoring of AMI. Table 1 summarizes the aptamer sequence used in the development of the electrochemical aptasensor for the detection of cTns. This sequence plays a critical role in the specificity and binding affinity of the aptasensors, enabling the accurate and reliable detection of cTns in AMI diagnostic applications.

**TABLE 1:** The aptamer sequence used in the electrochemical aptasensor for cTns detection.

S.No	Aptamer sequence	Biomarker	LOD	Ref.
1	5′‐SH‐TTTTTTCGTGCAGTACGCCAACCTTTCTCATGCGCTGCCCCTCTTA‐3′	cTnI	13 fg/mL	62
2	5′‐CGTGCAGTACGCCAACCTTTCTCATGCGCTGCCCCTCTTA‐3′	cTnI	0.18 pM	63
3	5′‐(SH)‐(CH_2_)6‐AGTCTCCGCTGTCCTCCCGATGCACTTGACGTATGTCTCACTTTCTTTTCATTGACATGGGATGACGCCGTGACTG‐3′	cTnI	0.05–500 ng/mL	64, 65
4	5′‐SH‐(C_6_)‐CGTGCAGTACGCCAACCTTTCTCATGCGCTGCCCCTCTTA‐MB‐3′	cTnI	0.95 pM	66
6	5‐NH_2_‐(CH_2_)6‐CGTGCAGTACGCCAACCTTTCTCATGCGCTGCCCCTCTTA‐3′	cTnI	1.04 pM	67
7	[ThiC6] CGTGCAGTACGCCAACCTTTCTCATGCGCTGCCCCTCTTA	cTnI and cTnT	2.58 ng/mL and 1.65 ng/mL	58
	And [ThiC6]ATACGGGAGCCAACACCAGGACTAACATTATAAGAATTGCGAATAATCATTGGAGAGCAGGTGTGACGGAT			

## 5 CHALLENGES AND FUTURE PERSPECTIVES IN APTASENSOR

Aptasensors, which utilize aptamers as biorecognition elements for the detection of various analytes, hold great promise for POC diagnostics and real‐time monitoring. However, several challenges remain in their widespread adoption and clinical translation. The short half‐lives of aptamers pose a significant challenge to the advancement in the field of aptasensor.^68^ Moreover, the current literature underscores a limited understanding of effective surface immobilization strategies for aptamers and highlights the need for a broader range of available aptamer sequences. Future advances may focus on hybridizing aptamers with nanomaterials to improve signal amplification, stability, and multiplexing capabilities. Furthermore, the incorporation of artificial intelligence and machine learning for data analysis could revolutionize the precision and adaptability of aptasensors in diagnostics.^69^, ^70^

## 6 SUMMARY AND CONCLUSIONS

POC diagnostics have revolutionized the early and precise detection of AMI, and rapid identification of cTn biomarkers is crucial for effective intervention. Optical signal transduction platforms have shown significant progress in enabling sensitive detection of cTn biomarkers. Colorimetric aptasensors using tetrahedral DNA nanostructures (TAPT‐TDNs) achieved a detection limit as low as 27 pg/mL, while a microfluidic paper‐based analytical device (μPAD) demonstrated dual detection ranges and an impressive detection limit of 0.0004 μg/mL. Fluorescent aptasensors based on graphene oxide (GO) platforms provided a detection range of 0.10–6.0 ng/mL, with a low detection limit of 0.07 ng/mL. Surface‐enhanced Raman scattering (SERS) platforms have emerged as a powerful diagnostic tool, achieving ultra‐low detection limits, including 100 aM (2.4 fg/mL) for cTnI in buffer solutions and 0.27 pg/mL in serum using ratiometric calibration. These advances underscore the potential of optical aptasensors for high‐precision AMI diagnostics.

Electrochemical aptasensors have made significant progress and have provided rapid, cost‐effective, and highly sensitive detection of cTnT. A ferrocene‐modified silica nanoparticle‐based aptasensor achieved an ultralow detection limit of 1.0 pM (24 pg/mL) for cTnI, while an ultrasensitive extended gate ion‐sensitive field effect transistor (EGISFET) platform detected cTnI within 5 min, with a detection limit of 0.3 pg/mL. A sandwich‐type electrochemical aptasensor utilizing dendritic platinum‐copper alloy nanoparticles and graphene‐like ultrathin nanosheets demonstrated an exceptional detection limit of 2.3 fg/mL and a wide detection range of 0.01 pg/mL to 500 ng/mL. Furthermore, a multiplexed electrochemical sensor platform achieved simultaneous detection of validated cTnT (LOD: 1.65 ng/mL) and cTnI (LOD: 2.58 ng/mL) in clinical samples. Other approaches, such as surface plasmon‐enhanced electrochemiluminescence (SPEECL) sensors and impedimetric sensors, have achieved detection limits as low as 0.75 fg/mL and 0.055 pg/mL, respectively, offering robust and clinically viable solutions.

In conclusion, aptasensor‐based POC diagnostics represent a significant step forward in AMI care, enabling rapid, sensitive, and accessible detection of cTn biomarkers. These platforms, with their ultralow detection limits and rapid analysis times, have the potential to revolutionize early diagnosis and prompt intervention.

## CONFLICT OF INTEREST STATEMENT

The authors declare that there are no conflicts of interest.

## Data Availability

Data sharing is not applicable to this article as no new data were created or analyzed in this study.
